# Combining pigment analysis and imaging microscopy to identify seasonal patterns in biomass and diversity of plankton

**DOI:** 10.1093/plankt/fbag056

**Published:** 2026-07-16

**Authors:** Nicole Corbett, Alice C Ortmann, Emmanuel Devred

**Affiliations:** Bedford Institute of Oceanography, Fisheries and Oceans Canada, 1 Challenger Drive, Dartmouth, Nova Scotia, B2Y 4A2, Canada; Bedford Institute of Oceanography, Fisheries and Oceans Canada, 1 Challenger Drive, Dartmouth, Nova Scotia, B2Y 4A2, Canada; Bedford Institute of Oceanography, Fisheries and Oceans Canada, 1 Challenger Drive, Dartmouth, Nova Scotia, B2Y 4A2, Canada

**Keywords:** flow imaging microscopy (FIM), high-performance liquid chromatography (HPLC), monitoring, methodology, environmental drivers

## Abstract

Multi-decade ocean monitoring of plankton is important to distinguish between short-term variability due to environmental drivers and longer-term changes due to climate stressors. Traditional methods for plankton enumeration are time consuming, have long lag times, and preservation may result in cell shrinkage. Fluorometric measurements of Chlorophyll-*a* concentrations exclude pigment separation, which is required to identify and quantify major taxonomic groups. To address these challenges, flow imaging microscopy (FIM) and high-performance liquid chromatography (HPLC) pigment analysis were combined to optimize the detection of plankton biomass seasonal patterns and their environmental drivers in the Bedford Basin, Nova Scotia, Canada. Eleven plankton sub-groups were detected, three of which overlapped between methods (pigmented dinoflagellates, diatoms and euglenoids). The HPLC was able to detect small cells (e.g. nano-diatoms), while FIM added higher taxonomic resolution and detection of non-pigmented cells, including two of the highest biomass groups, ciliates and non-pigmented dinoflagellates. Hierarchical clustering of the composite community dataset revealed four communities: C1-diatom/cryptophytes, C2-ciliates/chlorophytes, C3-dinoflagellates/haptophytes and C4-diverse community, which were influenced by seasonal cycles associated with nutrient replenishment and stratification. The complementary approaches increased monitoring efficiency of plankton population dynamics, which is essential for understanding the marine ecosystem and potential impacts of anthropogenic climate change.

## INTRODUCTION

Multi-decade monitoring of ocean waters has become increasingly important as ocean warming and climate change effects are being observed globally (Bindoff *et al.,* 2019; Silvy *et al.,* 2020). Consistent, repeated sampling is required to distinguish between short-term variability (days, seasons) due to environmental drivers and longer-term changes (years, decades) due to climate or anthropogenic stressors (Henson *et al.,* 2021). Picoplankton (0.2–2 μm), nanoplankton (2–20 μm) and microplankton (20–200 μm) (Sieburth *et al.,* 1978), all collectively referred to as plankton in this study, are a vital area of focus in terms of monitoring the effects of changing oceanic conditions as they act as primary and secondary producers, providing sustenance to higher trophic levels, and contribute to biogeochemical cycling of essential nutrients (Dinasquet *et al.,* 2017). Phytoplankton act as indicators for changing conditions such as temperature and nutrients, as their rates of production are directly influenced by these abiotic factors (Li and Harrison, 2008). Plankton such as diatoms and dinoflagellates play a key role in exporting carbon from the surface to the ocean depths (Nair *et al.,* 2008) and thus, impacts on plankton by climate change are predicted to cause significant changes in the marine ecosystem (Lotze *et al.,* 2019; Heneghan *et al.,* 2023).

Traditional methods for plankton identification and biomass estimation via light microscopy (counting and imaging of individual cells) and Chlorophyll-*a* (Chl-a) concentrations (solvent extraction and fluorometry) provide a baseline for long-term monitoring datasets globally (Strickland, 1960; Michaels and Knap, 1996). Despite providing essential standards for monitoring, microscope counts are time consuming, often involving a long lag time between sample collection and interpretation. The time, and the need for significant taxonomic expertise, limits the number of samples that can be analysed (Karlson *et al.,* 2010). If additives are used to preserve samples as a strategy to cope with increasing demand, cell shrinkage can occur, which introduces bias in biovolume estimates (Álvarez *et al.,* 2014). Chl-a monitoring requires filtering seawater and, due to the lack of an effective strategy for pigment separation and quantification, does not give an accurate depiction of species diversity and abundance. Chl-a content varies in different types of phytoplankton, so concentrations cannot be directly linked to biomass.

Flow imaging microscopy (FIM) is an improvement on the scope of work over the traditional methods mentioned above as it combines digital imaging, microscopy and flow cytometry to allow for the rapid measurement and enhanced analysis of plankton dimensions and abundance (Sieracki *et al.,* 1998). The increased efficiency of FIM allows for enumeration of more cells per sample and provides quantitative data such as cell count per volume. The ability to run samples “live” without preservation, improves biomass estimates. High-performance liquid chromatography (HPLC) advances traditional pigment analysis based solely on Chl-a, due to its increased sensitivity and ability to rapidly separate over 40 pigments, providing insight into phytoplankton taxonomic composition and abundance (Jeffrey *et al.,* 1999). Once the pigments have been separated, ratios of marker pigments associated with specific plankton groups are used to identify pigmented plankton present in a sample (Nair *et al.,* 2008). HPLC provides quantitative analysis based on the intensity of the peaks, which correlate with the amount of pigment detected in the sample.

The Bedford Basin is a 17 km^2^ embayment of the Atlantic Ocean in Halifax, Nova Scotia, Canada with limited freshwater influence from the Sackville River and runoff from the nearby watershed (Li and Harrison, 2008). The Bedford Basin has a long narrow entrance that contributes to a flushing time of 3 months for the entire basin and net seaward flow for surface waters (Shan and Sheng, 2012). The Bedford Basin is a high-traffic area frequented by commercial ships, recreational boats, Canadian Coast Guard (CCG) and Royal Canadian Navy vessels. Due to this and exponential growth of the surrounding communities, the Bedford Basin and the Halifax Harbour have experienced numerous sources of pollution. For example, untreated wastewater from Halifax and surrounding communities was historically dispensed directly into the harbour at a rate of 181 million L per day (Halifax Legacy Content, 2011). While this was rectified in 2008 (Halifax Legacy Content, 2011), wastewater releases still occasionally occur, most recently in April 2025 (Hoffman, 2025). Anthropogenic influences alter the environmental conditions present in Bedford Basin, particularly nutrient concentrations, which, in turn, impact the plankton community.

**Fig. 1:**
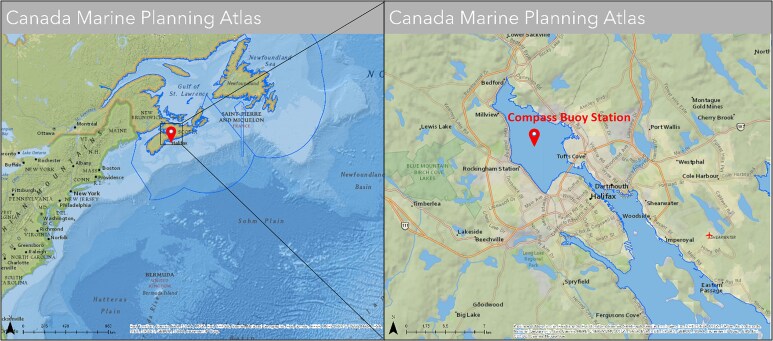
Map of location of the Compass Buoy Station (44° 41′ 37″ N, 63° 38′ 25″ W) where samples were collected within the Bedford Basin, Nova Scotia, Canada. The map on the left shows the location of Nova Scotia relative to the eastern coast of North America, with the map on the right showing the station location within the Bedford Basin. Maps created using the Marine Planning Atlas.

Since 1991, Fisheries and Oceans Canada’s (DFO) Bedford Basin Monitoring Program (BBMP) has conducted weekly measurements of the environmental and biological conditions at a set location referred to as the Compass Buoy Station (Fig. 1) (Mitchell *et al.,* 2002). This location was selected due to it being the deepest point of the Bedford Basin (71 m), and its close proximity to the Bedford Institute of Oceanography (BIO) allowed for convenient and consistent monitoring of seasonal and annual fluctuations representative of the Bedford Basin and the ocean surrounding Eastern Canada (Li, 2013). In addition to supporting research within DFO, the collected data also support academic research and studies led by the provincial government, and may be used for the management of marine resources and the protection of the ocean (Therriault *et al.,* 1998).

In this paper we present a case study showing how two methodologies, FIM and HPLC analysis of pigments, can be used together to optimize the detection of plankton biomass seasonal patterns and their environmental drivers over a multiyear dataset collected from the Bedford Basin.

## METHODS

### Sample collection and processing

Water column samples were collected from the Bedford Basin in Nova Scotia, Canada approximately once per week as part of the BBMP’s sampling at the Compass Buoy station using the CCGS *Sigma T* from 2 May 2018 to 16 March 2020. Physiochemical properties of the water column were collected using a Sea-Bird (SBE) Scientific SBE 25 Conductivity–Temperature–Depth (CTD) system. Deployment of the CTD involved lowering it from the vessel using a winch to 10 m depth for a 3-min soak period designed to flush the plumbing and stabilize the sensors, raising the system back to surface and then lowering it to within 2–3 m from the bottom at a speed of ~30 m/min. Water samples were collected at three standard depths (1, 5 and 10 m) using Niskin bottles mounted on the winch wire and a messenger closing system. Once recovered, the water from each Niskin bottle was stored in separate plastic 5 L carboys, placed in a cooler and kept in the dark until they were transferred to the laboratory at BIO where they were subsampled for analysis.

Carboys were subsampled for nutrient, FIM and HPLC analysis. Inorganic nutrients were measured using a SEAL Analytical AA3 continuous segmented flow autoanalyzer (SEAL Analytical Inc., Mequon, WI) using standard methods (Becker *et al.,* 2020). Silicate (Si), phosphate (PO_4_^−3^), ammonium (NH_4_^+^), nitrite (NO_2_^−^) and nitrite + nitrate (NO_2_^−^ + NO_3_^−^) were measured directly, while NO_3_^−^ was calculated by subtracting NO_2_^−^ from the NO_2_^−^ + NO_3_^−^ value.

### Plankton analysis

#### Plankton enumeration using FIM

Subsamples were processed shortly after collection using the FlowCam® 8000 (Fluid Imaging Technologies Inc., Scarborough, ME) dynamic imaging particle analyser (Cobanli *et al.,* 2022). Water samples from each depth were mixed by inversion and then filtered through mesh corresponding to the objective size (600 μm for the 4× objective, 80 μm for the 10× objective) to prevent clogging. All samples were processed using both the 4× and 10× objectives and both the Trigger and Autoimage modes to ensure a broad size range was covered (~10–1500 μm equivalent spherical diameter or ESD, with 10–80 μm particles detected with the 10× objective and >80 μm particles detected with the 4× objective). The optical system was focused using microbeads (50 and 25 μm for 4× and 10× objectives, respectively) to allow for clear imaging. Captured images from the Autoimage mode were classified using the image recognition software Visual Spreadsheet® version 4. Libraries were created for each group of particles using a collection of representative images that the software used to automatically classify each image captured into their corresponding groups (Camoying and Yniguez, 2016). Each classification was then visually reviewed to correct for potential misclassification.

The abundance data in cells mL^−1^ were exported to a spreadsheet, manually processed to combine 4× and 10× groups from the same organism captured under both objectives and filtered to remove non-living groups (beads, debris). After the data were filtered, 37 individual plankton groups remained. An average biovolume for each group was determined from the libraries based on standard geometric shapes (e.g. prolate spheroid, sphere, cylinder). For multicellular chains, the average biovolume was computed for the whole chain. Average biovolumes were used to determine a carbon-to-cell estimate (pg C cell^−1^), which allowed the abundance data to be converted into carbon biomass measured in microgram of carbon per litre (μg C L^−1^) (Menden-Deuer and Lessard, 2000).

Individual groups of plankton were identified to the lowest taxonomic level possible, typically genus, using several references: Identifying Marine Phytoplankton (Tomas, 1997), Plankton Monitoring Program in the Bedford Basin, 1991–1997 (Li *et al.,* 1998), World Register of Marine Species (WoRMS Editorial Board, 2026) and Nordic Microalgae (Torstensson *et al.,* 2024). Trigger mode, which only captures particles that emit fluorescent light, such as those emitted by excited phytoplankton photopigments, was used to distinguish pigmented dinoflagellates from non-pigmented dinoflagellates, with confirmation from the literature (Lessard and Swift, 1986; Bockstahler and Coats, 1993; Jeong *et al.,* 2010). Once identified, the 37 individual groups were further organized into the following 5 broader groups of plankton: ciliates (5), pigmented dinoflagellates (7), non-pigmented dinoflagellates (6), diatoms (18) and euglenoids (1).

#### Phytoplankton pigments and chemotaxonomy analysis

Pigment concentrations were measured using HPLC (Beckman and Dickson Gold from 1996 to 2013 and Agilent 1200 from 2014 to 2021). Water from each depth was vacuum filtered at low pressure (<10 dpi) through 25 mm Whatman® GF/f filters shortly after collection. The volume of water filtered varied from 0.25 to 1 L based on visual inspection of the filter, to ensure that colour was visible and thus there would be adequate material for pigment analysis. The filters were stored at −80°C until processing on the HPLC system. Details on the HPLC method to extract and measure pigment concentrations can be found in Head and Horne (1993) and Stuart and Head (2005).

For this study, 10 pigments were selected to identify phytoplankton taxonomic groups in water samples (Jeffrey and Vesk, 1997; Roy *et al.,* 2011). These pigments were chlorophyll-b (chl-b), chlorophyll-c_3_ (chl-c_3_), chlorophyll-c_1,2_ (chl-c_1,2_), fucoxanthin (fuco), peridinin (peri), zeaxanthin (zea), alloxanthin (allo), 19′-butanoyloxyfucoxanthin (19′-but), 19′-hexanoyloxyfucoxanthin (19′-hex) and diadinoxanthin (diadino). Initialization of phytoplankton groups and their pigment signatures was carried out using samples collected at Halifax-2 (HL_02), a high-frequency monitoring station located ~30 km offshore from the entrance to Halifax Harbour. As this site has phytoplankton taxonomy obtained from light microscopy counts along with HPLC data, phytoplankton groups derived from pigments could be validated. Additionally, because of the seaward flow of surface water from Bedford Basin (Shan and Sheng, 2012), HL_02 tends to have similar plankton communities as those found within the Basin. From the samples at HL_02, nine taxonomic groups (with their diagnostic pigment) were identified: dinoflagellates (peri and chl-c_1,2_), cryptophytes (allo and chl-c_1,2_), haptophytes (prymnesiophytes including coccolithophores; chl-c_3_, fuco, diadino and chl-c_1,2_), *Phaeocystis* spp. (chl-c_3_, fuco, 19′-hex, diadino and chl-c_1,2_), diatoms (chl-c_3_, fuco, diadino and chl-c_1,2_), dictyophytes (chl-c_3_, fuco, 19′-but, diadino and chl-c_1,2_), chlorophytes (chl-b and zea), euglenoids (chl-b and diadino) and cyanobacteria (zea) (Table SI).

The phytoclass R package (Hayward *et al.,* 2023), along with the pigment ratios derived from HL_02 samples, were used to derive the biomass of each phytoplankton group based on its pigment signature. The *phytoclass*() function is a statistical approach that simultaneously optimizes the pigment signature of the nine taxonomic groups considered and their biomass in water samples to retrieve the total concentration of each pigment in the samples. While the current study is focused only on the time frame between May 2018 and March 2020, the HPLC time series used to derive the taxonomic groups at the compass station is much longer (1995–2025). To increase the robustness of the pigment concentration estimates, *phytoclass*() was applied to the whole 31-year-long dataset. Before applying the *phytoclass*() function, samples were clustered into four groups sharing similar pigment signatures using the R *kmean*() function (Table SII) as recommended in Hayward *et al.* (2023). The pigment concentrations and biomass of each phytoplankton taxonomic group were calculated for each cluster. Samples corresponding to the FIM samples were extracted from the entire dataset, and the biomasses (μg Chl-a L^−1^) of the nine taxonomic group were converted into carbon biomass (μg C L^−1^) using C-to-Chl-a ratios previously established in the literature (Finenko *et al.,* 2003; Sathyendranath *et al.,* 2009), to allow for direct comparison with the FIM data.

### Data analysis

For the majority of dates within the 2-year period of this study, samples were analysed with both HPLC and FIM. Due to unavailability of the instrument, samples were not processed using FIM between 8 August 2019 and 4 September 2019, and HPLC analysis were missing for 10 samples (3 May 2018—10 m, 19 September 2018—1 m, 21 and 28 November 2018—10 m, 9 and 23 January 2019—1 m and 5 m, 30 January 2019—10 m, 9 January 2020—10 m) due to insufficient pigment extraction. Seasons were defined by National Research Council Canada (National Research Council Canada, 2025) start dates as follows: Spring (20 March), Summer (21 June), Autumn (23 September), Winter 2018/2019 (21 December) and Winter 2019/2020 (22 December). Each of the three depths sampled (1, 5 and 10 m) were treated as independent observations for clustering and correlation analysis where methods were being compared.

Spearman’s rank correlations were performed using the R base package to determine if the three groups (pigmented dinoflagellates, euglenoids and diatoms) that overlap between FIM and HPLC were correlated, as well as for comparing the total FIM and total HPLC biomass. Spearman’s rank correlation was chosen as the raw data met the following assumptions: the variables were numeric, and there was a monotonic relationship (confirmed with scatterplot and Kendall’s Nonparametric Test for Monotonic Trend).

A composite community dataset for total biomass was created which included the non-overlapping groups obtained from the HPLC analysis (cryptophytes, chlorophytes, dictyophytes, haptophytes, cyanobacteria and *Phaeocystis* spp.) and the non-overlapping groups from FIM (ciliates and non-pigmented dinoflagellates), as well as the shared groups (diatoms, euglenoids and pigmented dinoflagellates) taken from the HPLC data. Prior to clustering, the two datasets were fourth-root-transformed and Bray–Curtis dissimilarities were calculated. The base R stats package (R Core Team, 2025) was used to calculate cophenetic distances (*cophenetic*() function) and the cophenetic correlation (*cor*() function), which calculated the Pearson product moment correlation between the original Bray–Curtis distances and the cophenetic distances. The selection of clusters in the hierarchical clustering was validated by performing a silhouette width test in R using the *fviz_nbclust*() function from the factoextra R package (Kassambara and Mundt, 2020). Cluster selection was supported by a PERMANOVA test using the *adonis2*() function from the vegan package (Oksanen *et al.,* 2026). A SIMPROF test was also performed in PRIMER v6 & PERMANOVA + (Anderson, 2001; Clarke and Gorley, 2006). The dendrogram was generated using the *fviz_dend*() function also from the factoextra R package (Kassambara and Mundt, 2020).

To determine the impact of environmental drivers on plankton biomass, distance-based linear modeling (dbLM) was performed using seven physiochemical parameters (NH_4_^+^, NO_3_^−^, NO_2_^−^, PO_4_^−3^, Si, salinity and temperature), along with Julien day and depth using the statistical software PRIMER v6 & PERMANOVA + (Anderson, 2001; Clarke and Gorley, 2006) with AICc (Corrected Akaike’s Information Criterion) selection criteria and a step-wise procedure. To determine significant differences in the values of the environmental factors among clusters, linear mixed models (LMMs) were applied with Julian date as a random factor using lme4 (Bates *et al.,* 2015) and lmerTest (Kuznetsova *et al.,* 2017). To compensate for multiple tests, *P*-values were adjusted (Benjamini–Hochberg) using emmeans package (Lenth and Piaskowski, 2026). The Tukey Honestly Significantly Different (Tukey HSD) was used to identify which clusters differed. The maps were generated using the Canada Marine Planning Atlas (Fisheries and Oceans Canada, 2024). All remaining plots were created in R using ggplot2 (Wickham, 2016).

## RESULTS

### Seasonal biomass patterns in Bedford Basin

**Fig. 2:**
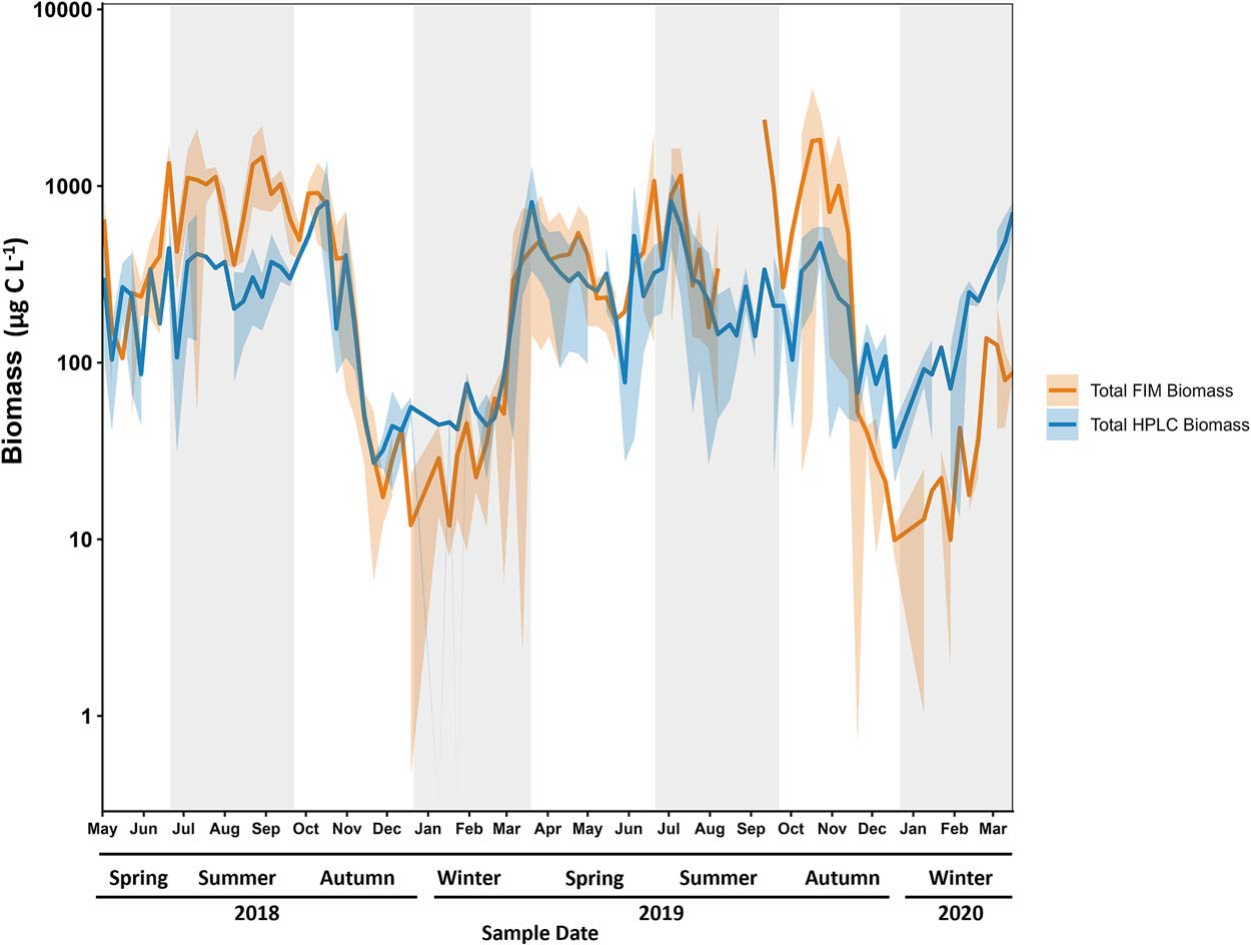
Total HPLC biomass and total FIM biomass over the time series (May 2018–March 2020). Raw data were averaged across all depths for each date with 95% confidence intervals overlayed in shading and displayed on a logarithmic scale. Grey shading indicates seasons.

Total FIM and total HPLC biomass were compared to determine if the two methods detected congruent seasonal plankton patterns in the Bedford Basin (Fig. 2). Overall, the FIM and HPLC biomass follow similar trends throughout the time series with total biomass lowest from mid-November to February of both sampling years and averages 2.4-fold higher in 2019–2020 (94.50 ± 23.41 μg C L^−1^) than in 2018–2019 (39.90 ± 9.44 μg C L^−1^). Total biomass also began to increase a month earlier in the 2019–2020 winter (January/February) than the previous winter (February/March). Higher total biomass was detected throughout the remainder of the months, with average concentrations of 460.41 ± 125.40 μg C L^−1^ and highest peaks in September/October, end of March and July. FIM total biomass tended to be higher than the HPLC total biomass for the spring, summer and autumn months and lower than HPLC total biomass for the winter months. This was reflected in a Spearman rank correlation test that determined the two methods to be positively correlated at rho = 0.69, which was considered statistically significant (*P*-value < 0.05, *n* = 262).

**Fig. 3:**
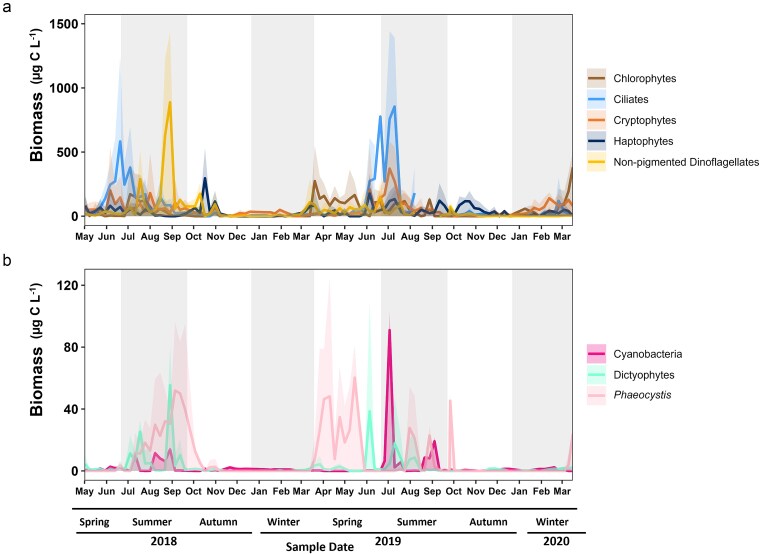
Seasonal biomass patterns of high biomass groups (**a**, over 100 μg C L^−1^) and low biomass groups (**b**, <100 μg C L^−1^). Raw data were averaged across all depths for each date with 95% confidence intervals overlayed in shading. Grey shading indicates seasons.

Of the five plankton groups detected with FIM, three overlapped with HPLC-detected groups (pigmented dinoflagellates, diatoms and euglenoids), with ciliates and non-pigmented dinoflagellates being unique to FIM and haptophytes, cryptophytes, chlorophytes, dictyophytes, cyanobacteria and *Phaeocystis* spp. being HPLC specific. Distinct seasonal patterns were observed within the eight plankton sub-groups that were detected by only one method (FIM or HPLC), which were split into high biomass groups (chlorophytes, ciliates, cryptophytes, haptophytes and non-pigmented dinoflagellates) with maximum biomass values over 100 μg C L^−1^ (Fig. 3a) and low biomass groups (cyanobacteria, dictyophytes and *Phaeocystis* spp*.*) with maximum biomass values <100 μg C L^−1^ (Fig. 3b). Ciliate biomass peaked in June/July for both sampling years, with approximately 1.5-fold higher biomass detected in 2019 than in 2018. Similarly, cryptophyte biomass primarily peaked in spring (June) and summer (July) of both years with biomass nearly double in July 2019 compared to July 2018. For the haptophytes, biomass was notable in autumn of 2018 (October) and increased to half the amount in spring of 2019 (June). Smaller haptophyte peaks were detected again in late summer/autumn of 2019. In terms of non-pigmented dinoflagellates, there were several peaks in biomass in the summer and autumn months, with the highest biomass detected in September of 2018. Smaller peaks were detected in the spring and summer of 2019. Chlorophyte biomass peaked in July 2018 and again in March and September of 2019.

While the remaining three groups had 10-fold lower biomass, individual seasonal patterns were still detected and contributed to understanding the plankton in the Bedford Basin (Fig. 3b). Cyanobacteria clearly peaked in July of 2019, with smaller amounts of biomass detected in the summer of 2018 (August/September) and 2019 (September). Dictyophytes were also present in the summer of both years, peaking in July and September of 2018 and earlier in 2019 in June and July. *Phaeocystis* spp. biomass was present and sustained during the summer of 2018 and spring of 2019, reaching similar levels in September 2018, April/May 2019 and October 2019.

**Fig. 4:**
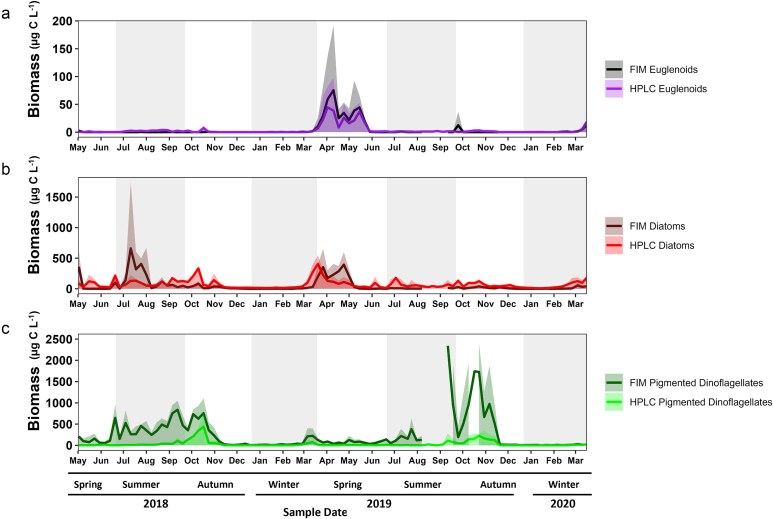
Seasonal biomass patterns of HPLC and FIM data for the three groups that overlap between the two methods: euglenoids **(a)**, diatoms **(b)** and pigmented dinoflagellates **(c)**. Raw data were averaged across all depths for each date with 95% confidence intervals overlayed in shading. Grey shading indicates seasons.

The three taxa that were common to both methods (euglenoids, diatoms and pigmented dinoflagellates) were compared to understand when and by how much the total biomass determined by FIM and HPLC differed and to assess whether the two methods detected comparable patterns in plankton biomass for these groups. Euglenoid biomass as determined by each method clearly overlapped at the same time of year, spring 2019, with the FIM biomass 1.5-fold higher than the HPLC biomass (Fig. 4a). Biomass estimates from the two methods were statistically correlated (*P*-value < 0.05, *n* = 262) with a weak positive correlation (Spearman’s rho = 0.33). The diatom biomass patterns identified by both methods mostly agreed in terms of the main peaks identified. The HPLC method determined higher biomass than FIM except during two events in July/August of 2018, where FIM biomass reached 4-fold higher levels and from April to May of 2019 (Fig. 4b). The FIM diatom biomass and the HPLC diatom biomass were determined to have a positive correlation at rho = 0.65 for the Spearman rank correlation, which was also statistically significant (*P*-value < 0.05, *n* = 262). In terms of pigmented dinoflagellates, the biomass detected by FIM was higher than the biomass detected by HPLC for the spring and summer months (Fig. 4c) but reached similar levels from autumn (October) until the start of the following summer. Biomass of pigmented dinoflagellates detected with FIM was high throughout autumn months, with the highest biomass detected in September and October of 2019, a 2- to 2.5-fold increase from the previous year. Spearman rank correlation determined the relationship between FIM and HPLC to be statistically significant (*P*-value < 0.05, *n* = 262) with a moderate correlation at rho = 0.56 for pigmented dinoflagellates.

### Plankton community distribution via clustering

Non-overlapping groups were all retained for generating the composite communities, while the shared groups were taken from the HPLC data. Although there was not complete overlap between FIM and HPLC for diatoms and pigmented dinoflagellates, the ability of HPLC to include small cells (Silwal, 2018), including the globally distributed nano-diatoms (Leblanc *et al.,* 2018), supported this approach. Hierarchical clustering analysis of the composite community was conducted using the group average linking method due to its higher cophenetic correlation value (0.64, *n* = 262) compared to other methods (e.g. single link, complete link, average link, Ward’s minimum variance and weighted average link). Clustering produced four clusters in three main groups at a resemblance level of 53 (height of 0.47) (Fig. S1). Within the four clusters, clusters C3 and C4 clustered together as more similar but distinct from both C1 and C2. PERMANOVA analysis found the four clusters to be statistically significant (*R*^2^ = 0.34, *F* = 44.10, *P*-value < 0.05, *n* = 262), and the SIMPROF test (significance level = 1%) provided additional statistical support for the cluster number.

**Fig. 5:**
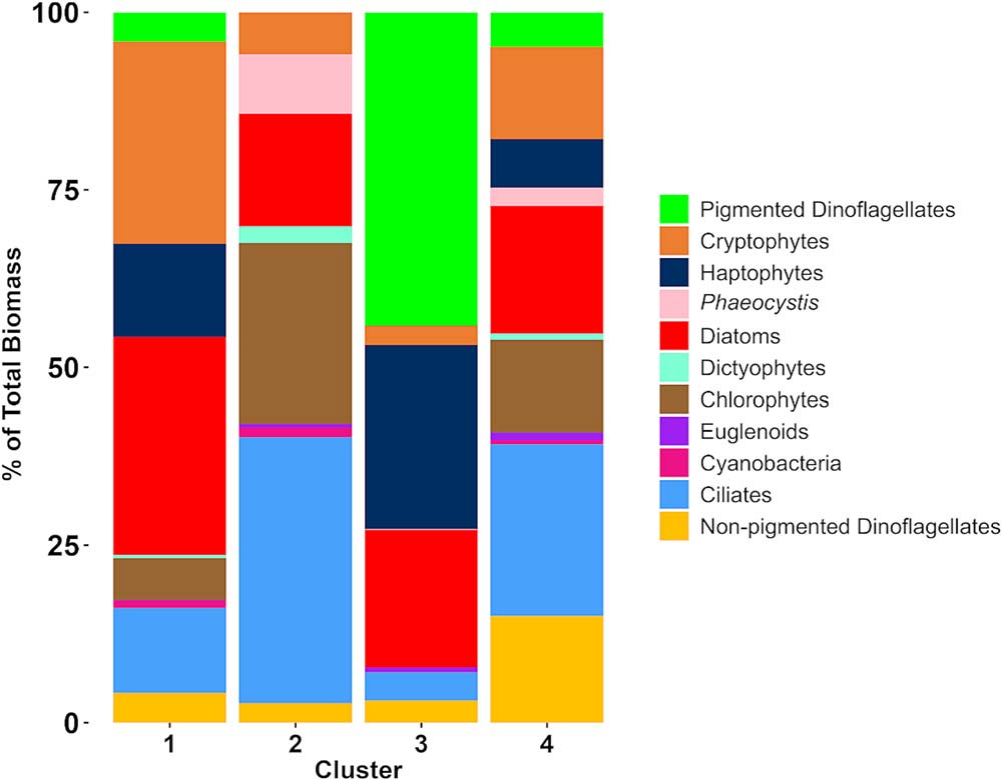
Stacked bar plots showing the average contribution in percent of total biomass of the 11 unique plankton groups for each of the four clusters determined from hierarchical clustering.

The distribution of the 11 groups of plankton identified by FIM and HPLC was unique in each cluster (Fig. 5). C1 was dominated by diatoms (37%) and cryptophytes (28%), with an equal proportion of ciliates (12%) and haptophytes (13%). C2 contained the smallest proportion of diatoms (16%), with large proportions of ciliates (37%) and chlorophytes (25%). Approximately half of C3 was composed of dinoflagellates, the majority of which were pigmented dinoflagellates (44%). This cluster also contained a large proportion of haptophytes (26%). C4 had the most equal distribution of taxa with similar proportion of ciliates (24%), non-pigmented dinoflagellates (15%), cryptophytes (13%), chlorophytes (13%) and diatoms (18%). This cluster was the most diverse, containing all 11 plankton groups.

**Fig. 6:**
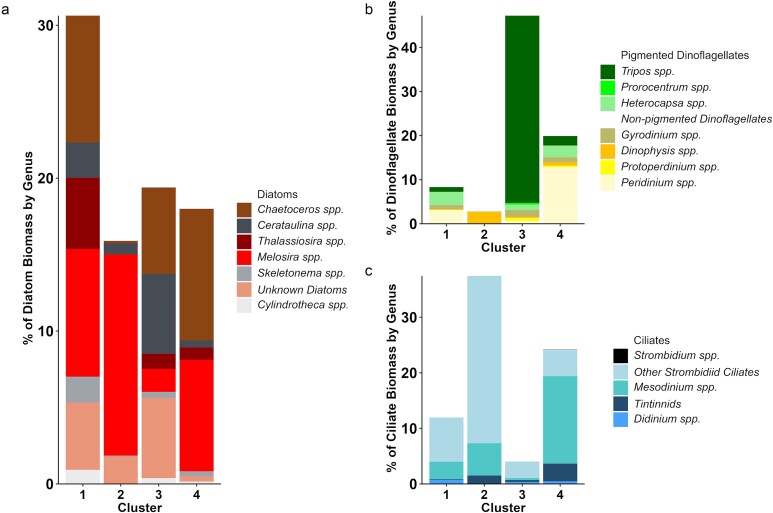
Stacked bar plots of FIM data showing the average percentage of genera identified from FIM for diatoms **(a)**, pigmented and non-pigmented dinoflagellates **(b)** and ciliates **(c)**. Bars add up to the contribution of each group shown in Fig. 5.

To understand the diversity of plankton present in these clusters, data collected by FIM can be used to provide greater taxonomic detail. The proportion of diatom biomass in each cluster is the extent of the information provided by the HPLC; however, from FIM, this proportion of diatom biomass can be further analysed to understand the diatom genera present (Fig. 6a). There were six diatom genera detected in the samples: *Chaetoceros* spp., *Melosira* spp., *Thalassiosira* spp., *Cerataulina* spp., *Skeletonema* spp. and *Cylindrotheca* spp. There was also a seventh group of diatoms that could not be confidently identified to the genus level of taxonomy and are presented as unknown diatoms. C1 and C3 both contained all seven diatom genera, where C1 was dominated by *Chaetoceros* spp. and *Melosira* spp., while C3 contained a larger proportion of *Cerataulina* spp. and unknown diatoms. C2 was markedly different, containing no *Thalassiosira* spp. or *Cylindrotheca* spp. and very little *Chaetoceros* spp. C4 contained a similar proportion of *Melosira* spp. and *Chaetoceros* spp.

Additional information about the composition of dinoflagellates can also be extracted from the FIM data. Three pigmented genera were detected in Bedford Basin: *Tripos* spp. (previously *Ceratium* spp.), *Heterocapsa* spp. and *Prorocentrum* spp., while four non-pigmented genera were present: *Peridinium* spp., *Gyrodinium* spp., *Protoperidinium* spp. and *Dinophysis* spp. (Fig. 6b). The genera and ratios of dinoflagellates varied widely across the four clusters. C1 contained an approximately equal proportion of *Heterocapsa* spp. and *Peridinium* spp., with very little *Tripos* spp. C2 contained only non-pigmented dinoflagellates, which largely consisted of *Dinophysis* spp. All seven dinoflagellates were detected in C3, with *Tripos* spp. heavily dominating. C4 was dominated by *Peridinium* spp.

Five groups of ciliates were detected using FIM, three of which were identified to genus: *Didinium* spp., *Mesodinium* spp., *Strombidium* spp., the other two will be referred to as Tintinnids and Other Strombidiid Ciliates (Fig. 6c). Other Strombidiid Ciliates were present in all four clusters; however, they were most prevalent in C2. C3 and C4 contained all five groups of ciliates, but where C4 was dominated by *Mesodinium* spp., C3 contained a relatively small proportion of all groups. C1 was dominated by *Mesodinium* spp., with a smaller contribution of Other Strombidiid Ciliates.

### Environmental drivers of plankton seasonal patterns

**Fig. 7:**
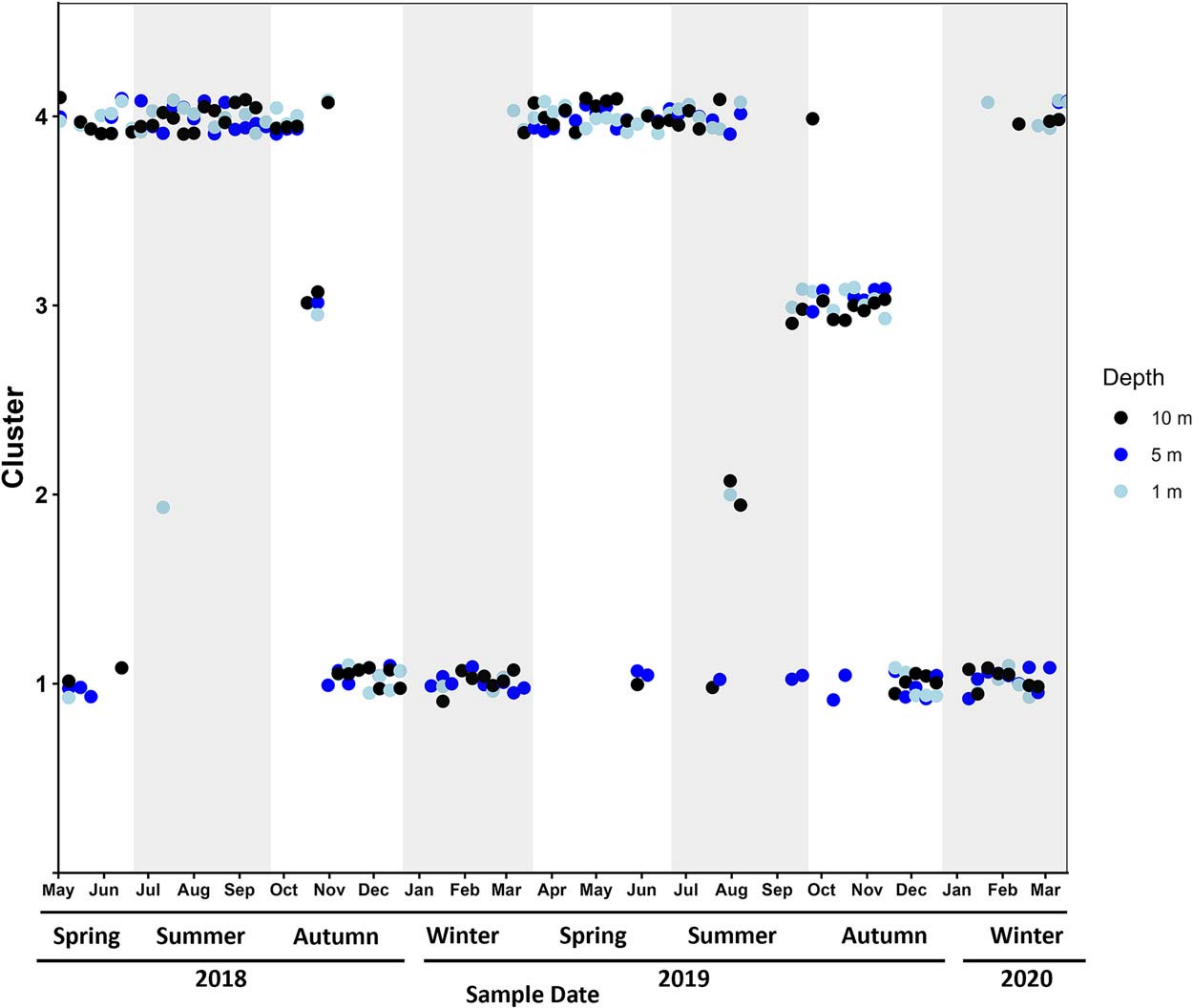
Indication of which cluster (C1-diatoms/cryptophytes, C2-ciliates/chlorophytes, C3-dinoflagellates/haptophytes, C4-diverse community) samples were assigned to, separated by depth (1, 5, 10 m), over the time series (May 2018–March 2020). Grey shading indicates seasons.

Hierarchical clustering did not separate the four plankton clusters by seasons; however, there was a season-dependent pattern with three main communities (C1, C3, C4) cycling throughout the year and one community (C2) detected in a small number of samples (Fig. 7). In most cases, a given cluster community occurred at all three depths (1, 5, 10 m) for any single date. Late autumn and winter samples were predominately identified as C1-diatom/cryptophyte communities, along with a small number of spring and summer samples. Spring and summer samples were mainly identified as C4-diverse communities, while late summer and early autumn samples represented C3-dinoflagellate/haptophyte communities. Three samples identified as C2-ciliate/chlorophyte communities were from the same time period in summer 2019, with one sample from summer 2018.

Seven physiochemical parameters—NH_4_^+^, NO_3_^−^, NO_2_^−^, PO_4_^−3^, Si, salinity and temperature—were evaluated alongside the biomass data to understand the impact of these environmental factors on the seasonal plankton cycles in Bedford Basin. Significant differences in the values of these environmental factors among clusters were identified through LMM and Tukey HSD analyses (Table I). The C1-diatom/cryptophyte cluster had the highest concentrations of nutrients and the coldest temperatures. The C2-ciliates/chlorophytes cluster and the C3-dinoflagellates/haptophytes cluster were characterized by the warmest conditions. Nutrient concentrations were similar across the C2-ciliates/chlorophytes, the C3-dinoflagellates/haptophytes and the C4-diverse community clusters. Salinity did not vary across clusters.

**Table I:** Mean and standard deviation of the seven environmental factors measured separated by cluster. Different letters indicate significant differences between clusters based on the Tukey HSD test, where the adjusted *P*-values (Benjamini–Hochberg) was <0.05. No letters indicate that clusters were not significantly different based on the LMM

	C1-diatoms/ cryptophytes	C2-ciliates/ chlorophytes	C3-dinoflagellates/ haptophytes	C4-diverse community
	*n* = 93	*n* = 4	*n* = 31	*n* = 134
NH4^+^ (μM)	2.99 (1.50)^c^	0.07 (0.04)^ab^	1.67 (1.36)^b^	0.65 (0.86)^a^
NO_3_^−^ (μM)	6.63 (2.73)^b^	0.00 (0.00)^a^	1.42 (1.81)^ab^	1.02 (1.91)^a^
NO_2_^−^ (μM)	0.21 (0.09)^c^	0.04 (0.00)^ab^	0.16 (0.13)^bc^	0.09 (0.11)^a^
PO_4_^−3^ (μM)	0.80 (0.24)^c^	0.12 (0.03)^a^	0.45 (0.20)^b^	0.33 (0.19)^ab^
Si (μM)	8.80 (3.56)^b^	0.32 (0.32)^a^	3.04 (2.20)^ab^	2.40 (2.91)^a^
Salinity	29.78 (0.75)	29.96 (0.37)	29.81 (0.71)	29.98 (0.69)
Temperature (°C)	4.76 (3.35)^a^	15.24 (4.05)^b^	12.22 (1.72)^ab^	8.58 (5.59)^a^

Distance-based linear modelling (dbLM) was used to identify which environmental parameters were impacting plankton biomass and in turn, driving seasonal plankton cycles in the Bedford Basin (Table SIII). Si, NO_3_^−^ and PO_4_^−3^ concentrations were determined to be highly correlated (*P* > 0.8); thus, NO_3_^−^ was used in the analysis and acted as a proxy for the two other nutrients. Marginal tests indicated the seven remaining variables should be included in the stepwise model building. NO_3_^−^, and by proxy Si and PO_4_^−3^, was by far the most important factor identified in the dbLM, explaining 22% of the variance in the biomass data (Pseudo-*F* = 73.19, *P*-value = 0.001). Temperature (Pseudo-*F* = 14.83, *P*-value = 0.001) and NH_4_^+^ (Pseudo-*F* = 10.13, *P*-value = 0.001) explained 4.2% and 2.8% of the variance, respectively. Julian day and depth and NO_2_^−^ were included in the model but explained less than < 2% of the variance each. Salinity was excluded from the final model, which explained 32% of the total variance with an *R*^2^_adjusted_ = 0.306.

**Fig. 8:**
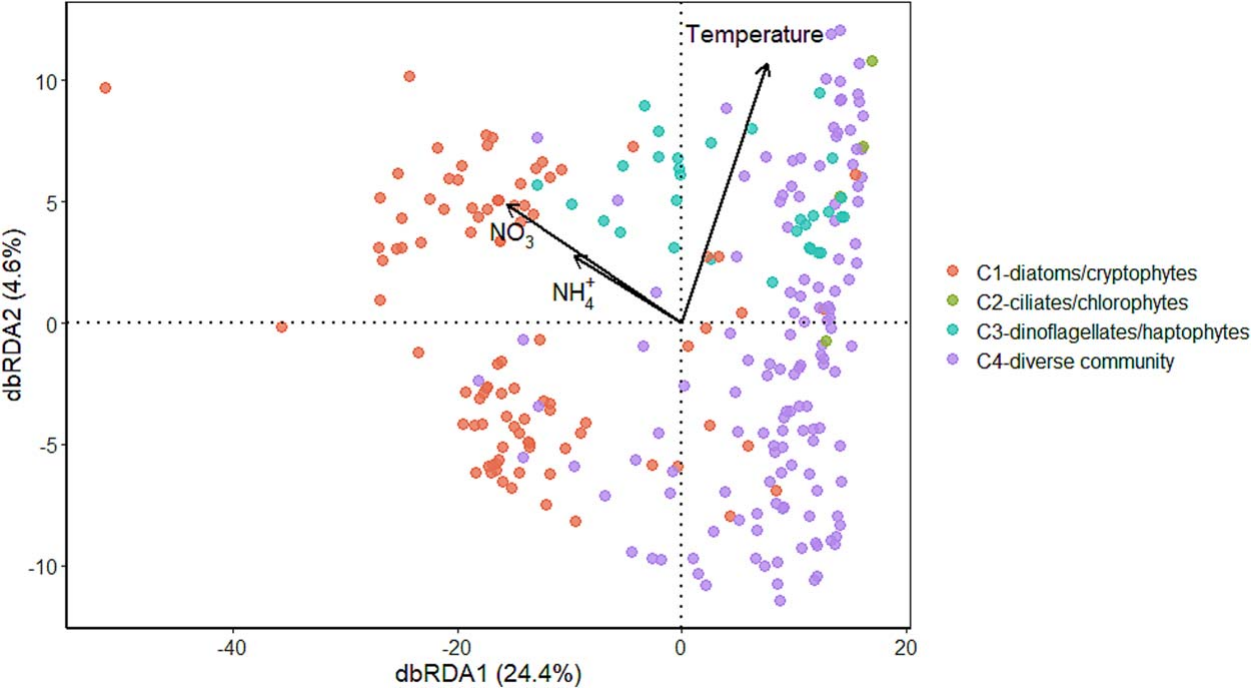
dbRDA plot of composite community plankton biomass based on Bray–Curtis dissimilarity. Samples are coloured by cluster and arrows indicated the direction and strength of the variables that explain the most variation in the biomass.

Distance-based redundancy analysis (dbRDA) was used to visualize how the three main factors may be related to the communities and the underlying seasonality. The arrow directions for the three environmental factors match what is expected for typical seasonal conditions in the Bedford Basin (Fig. 8) and what was observed in the mean environmental parameters for each community (Table I). C3-dinoflagellates/haptophytes (summer and autumn samples) and C2-ciliates/chlorophytes (summer samples) were most associated with high temperatures, while C1-diatom/cryptophytes (autumn and winter samples) were associated with higher nutrients, including NO_3_^−^ and NH_4_^+^. The C4-diverse community cluster (spring and summer samples) was associated with lower nutrient conditions. Both C1-diatom/cryptophytes and C4-diverse community were present across a broad range of temperatures. The dbLM indicated that the four communities generated are influenced by seasonal cycles as they tend to be dominated by samples from distinct seasons.

## DISCUSSION

### Method comparison of FIM and HPLC analysis

In this study, the total biomass measured by FIM and HPLC were found to be positively correlated, with the FIM biomass higher than the HPLC biomass for all seasons except for the winter of both sampling years. The moderately positive correlation between the two estimates reflects overlap in the plankton the two methods are detecting, while differences in their approaches result in some discrepancies in which plankton are accurately quantified by each method.

For instance, differences in the size ranges detected between the two methods will impact which groups are detected by FIM vs HPLC. Álvarez *et al.* (2011) recommended a combination of magnification and modes be used to effectively cover the spectrum of plankton, as done in this study. The 10× objective used with FIM has a theoretical minimum particle size of 2 μm (Yokogawa Fluid Imaging Technologies, 2026); however, only particles >~10 μm could be identified and quantified in this dataset. While higher magnification objectives (e.g. 20×) can be used with FIM, studies such as Graham *et al.* (2018) describe challenges associated with it, such as frequent clogging and an increased run time required as less sample volume can pass through the smaller flow cell per unit time. In this study, HPLC pigments were captured on a filter with a nominal pore size of 0.7 μm; thus, smaller cells could be identified using HPLC compared to FIM. Many of these smaller cells fell into the low-biomass phytoplankton groups detected only by HPLC. The effect of size differences was also observed in the different patterns in biomass estimated by HPLC compared to that estimated by FIM for diatoms, euglenoids and pigmented dinoflagellates. The FIM approaches inability to detect nanoplanktonic diatoms, such as *Minidiscus* spp., could explain differences between HPLC and FIM estimates in winter/early spring and autumn, when this genus is known to occur in relatively high abundance in the Bedford Basin (Stevens-Green *et al.,* 2025).

Other differences between biomass estimates from FIM compared to those from HPLC are driven by the use of images by FIM in contrast to using pigments with HPLC. In this study, the HPLC method used 10 of the 20 detected pigments as diagnostic pigments. If accurate diagnostic pigment ratios are determined for the sampling area (Simmons *et al.,* 2016), HPLC can distinguish taxa that are challenging to identify using imaging or microscopy techniques (Jeffrey *et al.,* 1999). Here, a nearby station, HL_02, was used to optimize the *phytoclass*() R function as both pigment data and taxonomy identified using light microscopy were available. Analysis of HL_02 data revealed nine main taxonomic groups, whose pigment signatures were determined from the literature (Roy *et al.,* 2011). The optimized parameters from HL_02 were applied to the Bedford Basin dataset. While these stations have been shown to share similar communities (Robicheau *et al.,* 2022), diagnostic pigment ratios for the enclosed bay could differ from the offshore station, introducing some error in the estimate of community composition from HPLC.

Additionally, the C-to-Chl-a ratios used in this study were selected from the literature and are considered conservative (Sathyendranath *et al.,* 2009). While they were determined from a broad range of trophic conditions including the Scotian Shelf, they are known to be highly variable and environmental parameters such as the specific light and nutrient levels in the Bedford Basin would impact the accuracy of the carbon estimates in this study. The complexity of pigments within different taxa of phytoplankton can also contribute to inaccuracies in identifying plankton. In this study, peridinin was used to identify dinoflagellates, but some dinoflagellates may contain alloxanthin and not peridinin (Zapata *et al.,* 2012), potentially resulting in an underestimation of dinoflagellate biomass. Conversely, this *phytoclass*() approach may cause an overestimation of the cryptophytes derived from the alloxanthin pigment. *Phaeocystis* spp. was included as a separate group in the *phytoclass*(), rather than including with the haptophyte group, due to their distinct pigment signature/pigment ratio and their important ecological role as they aggregate in large colonies that impact species succession, and carbon and nutrient cycling (Smith and Trimborn, 2024). Some of the potential errors in identification can be corrected by combining the FIM approach with HPLC.

The image-based approach of FIM adds to the HPLC data in two significant ways. The images provide higher taxonomic resolution, which enables monitoring of plankton of interest at the genus level, with some possibly identified to species. FIM methods that image all particles can also detect non-pigmented cells. The ciliates and non-pigmented dinoflagellates were two of the highest biomass groups detected in the Bedford Basin, which is likely why the total FIM biomass was higher than the total HPLC biomass, particularly in the summer/autumn months. A secondary method, like the FIM, is required to detect these abundant and functionally different groups (heterotrophs and mixotrophs) to understand their relationship with phytoplankton groups in the Bedford Basin.

### Seasonal cycle of plankton in the Bedford Basin

As a focus of a long-term monitoring program, the Compass Buoy Station has been well characterized and is considered to be representative of the Bedford Basin, although spatial variability may contribute to slightly different patterns in plankton over the whole Basin. Over the years, Chl-a measurements, flow cytometric analysis of small phytoplankton and microscope counts of Lugol’s fixed samples have provided insights into the seasonality of plankton (Mayzaud *et al.,* 1984; Li *et al.,* 1998, 2006; Li and Dickie, 2001; Robicheau *et al.,* 2022). The overarching patterns in plankton dynamics identified in this study agree with previous observations, with additional details into the dynamics of specific populations using the composite community, along with genus-level identifications. The Bedford Basin is well characterized by seasonal cycles of winter mixing at colder temperatures and a two-layer stratification at warmer temperatures (Li and Harrison, 2008). Stratification is established in spring (typically April), where the surface (~20 m) consists of a low-density layer that undergoes tidal exchange with the Atlantic Ocean/Scotian Shelf and a bottom saline layer that does not undergo circulation (Haas *et al.,* 2021). This persists into winter (November–February), until temperatures cool at which time the stratification is broken, and nutrients are replenished from vertical mixing (Haas *et al.,* 2021).

The dbLM results indicated that of the environmental parameters analysed, nutrient replenishment (particularly NO_3_^−^, PO_4_^−3^ and Si) was strongly associated with plankton community structure in the Bedford Basin. All plankton require inorganic nitrogen and PO_4_^−3^ for photosynthesis; however, diatoms are the only major group that requires Si at an approximately equal ratio to NO_3_^−^ (Li and Harrison, 2008). Production of new nitrogen by nitrogen-fixing cyanobacteria was identified annually in the Bedford Basin for the 4 years prior to this study (2014–2017), which may also impact plankton growth, in particular for haptophytes, due to their symbiotic exchange of fixed carbon for fixed nitrogen (Robicheau *et al.,* 2023).

Temperature was determined to be a secondary driver of plankton biomass, likely due to the impact temperature has on the stratification of the water and thus nutrient concentrations in Bedford Basin. A strong relationship between water temperature and picophytoplankton abundance has previously been identified in the Bedford Basin (Li, 2013). Small flagellates/picoplankton have been observed primarily during the summer/warmer temperatures (Mayzaud *et al.,* 1984; Li *et al.,* 2006; Robicheau *et al.,* 2022), which we now understand to be cyanobacteria, dictyophytes and *Phaeocystis* spp. While these groups had the lowest biomass, their presence or absence in the clusters provide insights into the population dynamics of plankton, such as potential grazing pressure by co-occurring groups like the Other Strombidiid Ciliates (Mayzaud *et al.,* 1984). NH_4_^+^ was determined as the tertiary environmental driver in this case study. NH_4_^+^ is known to influence phytoplankton species succession, particularly in stratified environments with organic matter recycling, as it can be more readily utilized for biomass synthesis than NO_3_^−^ and NO_2_^−^, which require reduction within plankton cells (Buchanan *et al.,* 2025). The high proportion of unexplained variance is likely due to other potential drivers that were not measured in this study such as light availability, episodic mixing events and grazing pressure. Notably, larger zooplankton grazers were not included in the current study.

Clustering revealed that plankton community composition, when paired with the forementioned environmental factors, provide detailed insight into the plankton diversity and seasonal cycling in Bedford Basin. Late autumn and winter samples, where temperatures were low and nutrients were high, were dominated by the C1-diatom/cryptophyte communities. This community was characterized by the largest proportion of diatoms, dominated by *Melosira* spp. and *Chaetoceros* spp., known to have consistent seasonal patterns of high abundance in the Bedford Basin (Robicheau *et al.,* 2022). The temperature decreases during this time, in addition to winds and storms, results in the breakdown of the vertical stratification, allowing for mixing and increased nutrients to reach the sunlit layers (Robicheau *et al.,* 2023). This, along with maximum light attenuation, likely initiated the diatom/cryptophyte bloom (Casault *et al.,* 2020). The “typical” timing of the diatom spring bloom that is known to occur in the Northwest Atlantic (Casault *et al.,* 2024) was observed in March–May of 2019. This spring bloom was accompanied by an increase in euglenoids, a clear temporal pattern observed by Robicheau *et al.* (2022) for each year between 2014 and 2017.

Following this bloom, in the spring/summer the C4-diverse community, ciliates like *Mesodinium* spp. and non-pigmented dinoflagellates like *Peridinin* spp. increased, which could be in response to the high diatom biomass, their major food source (Gifford, 1988). Non-pigmented dinoflagellates in particular have been found to have the largest grazing impact on bloom-forming diatoms (Sherr and Sherr, 2007). Temperature and nutrients were in the mid-ranges, and this well-mixed water promoted a high diversity with all 11 plankton groups present and a more even distribution of phytoplankton size classes, as previously reported (Li, 2002).

The C2-ciliates/chlorophytes were a rare sub-community that primarily occurred in the height of summer, which agreed with previous observations of chlorophyte peaks in July in the Bedford Basin (Robicheau *et al.,* 2022). While the ciliates were at their peak, the cryptophytes were significantly reduced during this time. Ciliates are known to feed on cryptophytes, not just for sustenance but also to retain and utilize their chloroplasts for photosynthesis (Johnson *et al.,* 2006; Qiu *et al.,* 2016)*.* This unique feeding strategy may explain the relationship observed between these two groups in this case study, which would not have been observed by using either FIM or HPLC in isolation. During this period of intense stratification, temperatures elevated to slightly higher than normal, and nutrients were lowest. NO_3_^−^ reached the lowest concentrations since 1997, and PO_4_^−3^ and NO_2_- reached the lowest levels on record (Casault *et al.,* 2020). Low nutrient conditions exacerbate the competition for resources and tend to favor smaller phytoplankton like cyanobacteria, *Phaeocystis* spp. and some dictyophytes with lower nutrient requirements and more efficient mechanisms at absorbing those nutrients (Raven, 1998; Litchman *et al.,* 2010; Burson *et al.,* 2018), as was the case in C2. There were no pigmented dinoflagellates present, and diatom biomass was lowest during this time, likely due to nutrient depletion (Haas *et al.,* 2021) and potential grazing by non-pigmented dinoflagellates like *Dinophysis* spp. While the other diatom genera had notable decreases during this time, biomass of *Melosira* spp. remained at similar quantities to the other clusters/times of the year. This nonseasonal presence was also documented in *Melosira nummuloides* by McLean *et al.* (1981), who describe a facultative chemoheterotrophic ability, particularly in polluted environments. The C2-ciliates/cryptophytes appear to represent an episodic event due to the high-temperature and low-nutrient conditions mentioned in Bedford Basin during that period, rather than a recurring dominant community.

Previous studies of the Bedford Basin have identified increases in pigmented dinoflagellates in autumn, likely in response to increased nutrients following a weakening in the vertical stratification of the water column (Li, 2014). A similar trend was observed in the composite plankton community, with pigmented dinoflagellates being one of the more dominate populations in C3-dinoflagellates/haptophytes. Using the additional analysis from FIM, these dinoflagellates were found to be dominated by *Tripos* spp., along with *Heterocapsa* spp. Similarly, the haptophytes have been observed to peak near the autumn Chl-a maximum (Robicheau *et al.,* 2022) as was observed in the C3 cluster community. Meanwhile, the ciliate population greatly declined, particularly *Mesodinium* spp.

## CONCLUSION

Our case study of the Bedford Basin demonstrated the utility of combining FIM and HPLC pigment analysis for monitoring plankton. While many studies (Jeffrey *et al.,* 1999; See *et al.,* 2005; Nair *et al.,* 2008; Roy *et al.,* 2011; Silwal, 2018) recommend using multiple methods for plankton enumeration, few have provided a detailed complementary approach to maximize the plankton taxa information using two distinct methods. The time series included a large number of samples, collected weekly for ~2 years, which helped ensure reliability of the comparison. When utilized together, FIM and HPLC approaches expanded the size spectrum of plankton measured to include picoplankton, nanoplankton and microplankton. The two methods were found to overlap in terms of detecting diatoms, pigmented dinoflagellates and euglenoids, which allowed for verification of the accuracy of each method and an understanding of the observed differences. HPLC was better able to characterize taxa that are difficult to do so using imaging techniques due to their small size and provides a high-level snapshot of the phytoplankton diversity present, while FIM can supplement biomass estimates with genus or species level information to provide a more in-depth understanding of the dynamics at the base of the food chain. Monitoring both pigmented (autotrophic) and non-pigmented (heterotrophic) plankton groups gives insights into the grazing behaviour and trophic relationships that would otherwise be missed. Plankton seasonal biomass patterns were determined for the 11 plankton groups that were primarily driven by nutrients (NO_3_^−^, Si and PO_4_^−3^), which can be explained by the strong physical influences (stratification, mixing) that occur annually in the Bedford Basin, that in turn, impacts the availability of nutrients. The complementary approach outlined allowed for an efficient and powerful high-frequency monitoring of plankton, providing an understanding of plankton population dynamics which is essential for understanding the marine ecosystem as a whole and potential impacts of anthropogenic climate change.

## Supplementary Material

Final_Rev1_JPR_Corbett_Supplemental_fbag056

## Data Availability

The data that support the findings of this study are available from the corresponding author upon request.
