# Supplementary material for: Combining pigment analysis and imaging microscopy to identify seasonal patterns in biomass and diversity of plankton
Source: J Plankton Res. 2026 Jul 16;48(4):fbag056. doi: 10.1093/plankt/fbag056 (PMC13374663; doi:10.1093/plankt/fbag056)
Supplement: Final_Rev1_JPR_Corbett_Supplemental_fbag056 [file final_rev1_jpr_corbett_supplemental_fbag056.pdf]

Supplemental Material

Combining pigment analysis and imaging microscopy to identify seasonal patterns in biomass and diversity of plankton

Nicole Corbett, Alice C. Ortmann*, Emmanuel Devred,

Fisheries and Oceans Canada, Bedford Institute of Oceanography, 1 Challenger Drive, Dartmouth, Nova Scotia, B2Y 4A2 Canada

Contents

Supplementary Tables SI, SII, SIII

Supplementary Figure 1

Table SI. Matrix of pigment to chl-a ratios for the nine taxonomic groups quantified in the current study. Ratios were derived for samples collected at HL_02 where microscopy data was used to ground truth the pigment analysis. Pigments included chlorophyll-a (chl-a), chlorophyll-b (chl-b), chlorophyll-$c_{1,2}$, (chl-$c_{1,2}$), chlorophyll-c3(chl-c_3), fucoxanthin (fuco), 19’-butanoyloxyfucoxanthin (19’-but), 19’-hexanoyloxyfucoxanthin (19’-hex), peridinin (peri), zeaxanthin (zea), alloxanthin (allo), and diadinoxanthin (diadino).

	**Chl-b**	**Chl-$c_{1,2}$**	**Chl-c_3**	**Fuco**	**Peri**	**Zea**	**Allo**	**19’-but**	**19’-hex**	**Diadino**
**Dinoflagellates**	0.00	0.20	0.00	0.00	0.79	0.00	0.00	0.00	0.00	0.00
**Cryptophytes**	0.00	0.05	0.00	0.00	0.00	0.00	0.60	0.00	0.00	0.00
**Haptophytes**	0.00	0.15	0.11	0.14	0.00	0.00	0.00	0.00	0.00	0.10
***Phaeocystis* spp.**	0.00	0.20	0.30	0.06	0.00	0.00	0.00	0.00	0.08	0.05
**Diatoms**	0.00	0.12	0.01	0.63	0.00	0.00	0.00	0.00	0.00	0.16
**Dictyophytes**	0.00	0.40	0.040	0.30	0.00	0.00	0.00	0.05	0.00	0.13
**Chlorophytes**	0.03	0.00	0.00	0.00	0.00	0.04	0.00	0.00	0.00	0.00
**Euglenoids**	1.20	0.00	0.00	0.00	0.00	0.00	0.00	0.00	0.00	0.40
**Cyanobacteria**	0.00	0.00	0.00	0.00	0.00	0.40	0.00	0.00	0.00	0.00

Table SII. Mean pigment concentration for the four clusters generated from HPLC samples collected between 1995 and 2025 that were used to generate biomass estimates. Pigments included chlorophyll-a (chl-a), chlorophyll-b (chl-b), chlorophyll-$c_{1,2}$, (chl-$c_{1,2}$), chlorophyll-c3(chl-c_3), fucoxanthin (fuco), 19'-butanoyloxyfucoxanthin (19'-but), 19'-hexanoyloxyfucoxanthin (19'-hex), peridinin (peri), zeaxanthin (zea), alloxanthin (allo), and diadinoxanthin (diadino). N=number of samples in each cluster.

Cluster	N	Chl-a	Chl-b	Chl-$c_{1,2}$	Chl-c_3	Fuco	19'-but	19'-hex	Peri	Zea	Allo	Diadino
**1**	1038	3.22	0.16	0.31	0.03	0.40	0.01	0.02	0.14	0.01	0.46	0.08
**2**	686	5.79	1.36	0.67	0.18	1.01	0.09	0.28	1.23	0.04	0.24	0.58
**3**	1514	5.45	0.21	0.57	0.07	1.44	0.03	0.04	0.16	0.02	0.15	0.24
**4**	648	5.73	0.21	1.14	0.14	2.53	0.02	0.04	0.20	0.01	0.14	0.48

Table SIII. Results from distanced based linear model including the marginal tests and stepwise sequential tests for Bray-Curtis dissimilarity of composite community biomass (n=262). AICc selection criteria was used to select the best model. SS(trace) = sums of squares of trace statistic, Pseudo-F = pseudo F-statistics, Prop = proportion of variance explained by each variable. Cumul = cumulative proportion of variance explained, Res-df = residual degrees of freedom, and VIF = variance inflation factor.

Marginal tests

Variable	SS(trace)	Pseudo-F	P-value	Prop.
Nitrate	43697	73.19	0.001	0.220
Salinity	2505.5	3.317	0.016	0.012
Temperature	19797	28.74	0.001	0.099
Ammonium	35495	56.47	0.001	0.178
Nitrite	16138	22.95	0.001	0.081
Julian Day	6247	8.43	0.001	0.031
Depth	3887	5.18	0.003	0.019

Sequential tests

Variable	AICc	SS(trace)	Pseudo-F	P	Prop.	Cumul.	Res.df	VIF
+Nitrate	1676.7	43697	73.19	0.001	0.220	0.21967	260	2.87
+Temperature	1664.2	8409	14.83	0.001	0.042	0.26194	259	2.67
+Ammonia	1656.2	5546	10.13	0.001	0.028	0.28982	258	2.12
+Depth	1652.9	2855	5.30	0.002	0.014	0.30417	257	1.13
+Julian Day	1650.8	2202	4.12	0.001	0.011	0.31524	256	1.84
+Nitrite	1649.9	1529	2.89	0.017	0.008	0.32293	255	1.62

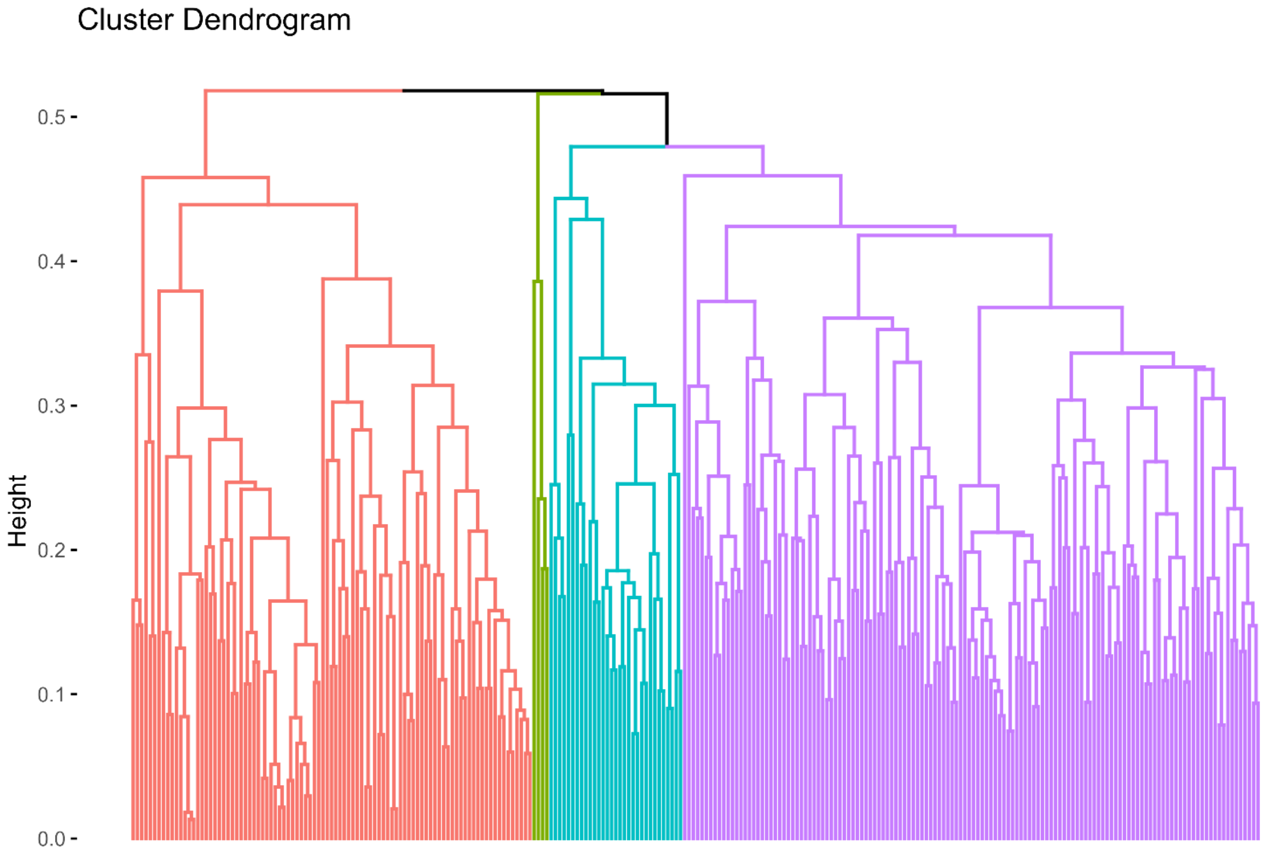

Fig S1. Hierarchical clustering via group average linking using composite community dataset (n=262). Raw data was 4th root transformed and a Bray-Curtis dissimilarity matrix was generated prior to hierarchical clustering. Four clusters were identified C1 (pink), C2 (green), C3 (blue) and C4 (purple).
